# Smart mandibular advancement devices for obstructive sleep apnea: a systematic literature review

**DOI:** 10.1007/s11325-024-03068-3

**Published:** 2024-06-18

**Authors:** Joshua Yang, Boudewijn R.A.M. Rosenmöller, Tom C.T. van Riet, Misha L. Tan, Faridi S. Jamaludin, Jean-Pierre T.F. Ho, Jan de Lange

**Affiliations:** 1 Harvard School of Dental Medicine, Boston, MA USA; 2 Department of Oral and Maxillofacial Surgery, Amsterdam University Medical Centre, Academic Medical Center (AMC), and Academic Centre for Dentistry Amsterdam (ACTA), University of Amsterdam, Meibergdreef 9, Amsterdam, 1105 AZ The Netherlands; 3 Department of Orofacial Pain and Dysfunction, Academic Centre for Dentistry Amsterdam (ACTA), Amsterdam, The Netherlands; 4 Information Specialist Medical Library, Amsterdam University Medical Centre, Academic Medical Center (AMC), Amsterdam, The Netherlands; 5 Department of Oral and Maxillofacial Surgery, Northwest Clinics, Alkmaar, the Netherlands

**Keywords:** Obstructive sleep apnea, Mandibular advancement device, Smart technology, Technological readiness level, Patient compliance, Feedback-controlled mandibular positioner

## Abstract

**Purpose:**

The goal of this review is to provide sleep physicians, dentists, and researchers with an evidence-based overview of the literature on smart mandibular advancement devices (MADs) for the treatment of obstructive sleep apnea.

**Methods:**

A systematic literature search was conducted by two blinded reviewers and an information specialist. A smart MAD was defined as any MAD with additional functionality besides mandibular protrusion. The bibliographic databases Medline, Embase, and Scopus were used to identify relevant publications. Studies were included if they described any stage of development of smart MADs. A total of 3162 titles and abstracts were screened for their relevance. In total, 58 articles were selected for full-text screening, 26 of which were included in this review.

**Results:**

The overall quality of the available literature was low. Most of the studies were observational, clinical or applied-research articles. The authors classified MADs into two main groups: passive and active. Passive MADs measured patient data, most commonly patient compliance. Active MADs adjusted protrusion of the mandible in response to patient data and were found in various phases of technological readiness (in development, demonstration, or deployment).

**Conclusion:**

Innovations in smart mandibular advancement devices most frequently track patient compliance. Devices measuring other health parameters and active, feedback-controlled, devices are increasingly reported on. However, studies demonstrating their added benefit over traditional methods remain sparse. With further study, smart mandibular advancement devices have the potential to improve the efficiency of obstructive sleep apnea treatment and provide new treatment possibilities.

**Supplementary information:**

The online version contains supplementary material available at 10.1007/s11325-024-03068-3.

## Introduction

Obstructive sleep apnea (OSA) is a highly prevalent condition in which repeated partial and/or complete upper airway collapse during sleep can lead to oxygen desaturation and sleep interruption [1, 2]. A systematic review [3] of the prevalence of sleep apnea described that, at an Apnea Hypopnea Index (AHI) ≥ 5 events per hour, OSA may be prevalent in at least 9% of the general population, and is significantly higher in men, the elderly, and more obese individuals [2].

A well-recognized treatment option for OSA is mandibular advancement device (MAD) therapy, which has proven to be successful in significantly reducing the AHI and alleviating OSA-related symptoms [4, 5]. Mandibular advancement devices work by increasing the oropharyngeal and hypopharyngeal airway during sleep, by keeping the lower jaw in a forward position, thereby preventing the upper airway from collapsing. Nonetheless, undesirable side effects of MAD usage — for example, malocclusion, temporomandibular joint disorders and low patient adherence — can lead to therapy failure [6].

To improve treatment modalities for OSA, technological innovations have been incorporated in various treatment modalities, e.g., continuous positive airway pressure (CPAP) [7], sleep position trainer (SPT) [8] and hypoglossal nerve stimulation [9]. These innovations have led to the development of the aforementioned appliances into what is frequently called “smart” appliances [7, 8]. In recent years, technological advances have also been introduced and applied in MADs [10, 11]. It has led to the development of a variety of “smart” MADs with a variety of functions and in different stages of development, from proof of concept to commercially available technology.

To our knowledge, there has been no evidence-based overview of the different initiatives to develop smart MADs, the stages of their development or their benefits to OSA treatment. This is important, as, due to their technical character, new developments in MADs may be published outside of routine clinical journals [12]. Therefore, it may be difficult for clinicians and researchers to track developments in the field. A review of these initiatives could also inspire researchers and industrial partners to develop and create novel modalities of MAD therapy. This review therefore aims to provide an evidence-based overview of the current literature on technological innovations in MADs and their readiness for clinical use.

## Materials and methods

### Information sources and search strategy

A systematic literature search was conducted with the collaboration of an information specialist (FJ). To identify all relevant publications, the bibliographic databases Medline (through PubMed), Embase, and Scopus were searched on 27th February 2023. The complete search strategies can be found in Tables 1, 2 and 3. To design the protocol of this review, the guidelines provided by the Preferred Reporting Items for Systematic Reviews and Meta-Analyses Extension for Scoping Reviews (PRISMA-ScR) and the Joanna Briggs Institute (JBI) [13, 14] were followed. The study was registered in the International Prospective Register of Systematic Reviews (PROSPERO) database with registration number CRD42023399655.

**Table 1:** Search strategies used in the databases. Search strategy used in PubMed

Concept	Search
1	(“Sleep Apnea Syndromes“[Mesh] OR “Snoring“[Mesh] OR “Dyspnea“[Mesh] OR “Apnea“[Mesh] OR apnea*[tiab] OR dyspnea*[tiab] OR dyspnoea*[tiab] OR OSA[tiab] OR OSAS[tiab] OR OSAHS[tiab] OR sleep-disordered breath*[tiab] OR hypopnea*[tiab] OR snoring*[tiab] OR breathing disorder*[tiab]) AND
2	(“Orthodontic Appliances“[Mesh] OR “Mandibular Advancement“[Mesh] OR “Occlusal Splints“[Mesh] OR mandibular advancement device*[tiab] OR mandibular advancement appliance*[tiab] OR mandibular advancement splint*[tiab] OR mandibular repositioning device*[tiab] OR MATRx plus[tiab] OR occlusal splint*[tiab] OR oral appliance*[tiab] OR oral device*[tiab] OR dental night guard*[tiab] OR intraoral device*[tiab] OR intraoral appliance*[tiab] OR intra-oral device*[tiab] OR intra-oral appliance*[tiab] OR dental device*[tiab] OR dental appliance*[tiab] OR oral appliance*[tiab] OR OAT[tiab] OR MAD[tiab] OR monobloc*[tiab] OR mono-bloc*[tiab] OR twin bloc*[tiab] OR bilateral thrust[tiab] OR duobloc*[tiab] OR bibloc*[tiab] OR duo-bloc*[tiab] OR bi-bloc*[tiab] OR midline traction[tiab]) AND
3	(“Oxygen Saturation“[Mesh] OR “Oximetry“[Mesh] OR “Oxygen“[Mesh] OR “Body Temperature“[Mesh] OR “Vital Signs“[Mesh] OR “Patient Compliance“[Mesh] OR “Treatment Adherence and Compliance“[Mesh] OR “Respiration“[Mesh] OR “Nocturnal Myoclonus Syndrome“[Mesh] OR saturation[tiab] OR oximetry[tiab] OR temperature[tiab] OR compliance[tiab] OR measure*[tiab] OR air flow*[tiab] OR airflow*[tiab] OR parameter*[tiab] OR breathing[tiab] OR periodic leg movement*[tiab] OR periodic limb movement*[tiab] OR vital sign*[tiab] OR oxygen[tiab] OR vital*[tiab] OR air[tiab] OR adher*[tiab] OR smart[tiab] OR intelligen*[tiab] OR computer*[tiab] OR sensor*[tiab] OR detector*[tiab] OR monitori*[tiab] OR auto*[tiab] OR remot*[tiab])

**Table 2:** Search strategies used in the databases. Search strategy used in EMBASE (OVID)

#	Searches
1	exp sleep disordered breathing/ or snoring/ or apnea/ or dyspnea/
2	(apnea* or dyspnea* or OSA or OSAS or OSAHS or sleep-disordered breath* or hypopnea* or snoring* or breathing disorder*).ti, ab, kf.
3	1 or 2
4	exp orthodontic device/ or exp mandibular advancement/ or exp dental therapeutic device/
5	((mandibular* or oral* or intraoral* or intra-oral* or dental* or occlusal*) adj3 (device* or appliance* or splint*)).ti, ab, kf.
6	(MATRx plus or dental night guard* or OAT or MAD or monobloc* or mono-bloc* or twin bloc* or bilateral thrust or duobloc* or bibloc* or duo-bloc* or bi-bloc* or midline traction).ti, ab, kf.
7	4 or 5 or 6
8	oxygen saturation/ or exp oximetry/ or oxygen/ or exp body temperature/ or vital sign/ or exp patient compliance/ or exp breathing/ or periodic limb movement disorder/
9	(saturation or oximetry or temperature or compliance or adher* or measure* or air flow* or airflow* or parameter* or breathing or periodic leg movement* or periodic limb movement* or vital sign* or oxygen or vital* or air or smart or intelligen* or computer* or sensor* or detector* or monitori* or auto* or remot*).ti, ab, kf.
10	8 or 9
11	3 and 7 and 10

**Table 3:** Search strategies used in the databases. Search strategy used in SCOPUS

Concept	Search
1	( TITLE-ABS-KEY ( apnea* OR dyspnea* OR osa OR osas OR osahs OR hypopnea* OR snoring* ) AND TITLE-ABS-KEY ( ( mandibular* OR oral* OR intraoral* OR intra-oral* OR dental* OR occlusal* ) AND ( device* OR appliance* OR splint* ) ) AND TITLE-ABS-KEY ( saturation OR oximetry OR temperature OR compliance OR adher* OR measure* OR airflow* OR parameter* OR breathing OR vital AND sign* OR oxygen OR vital* OR air OR smart OR intelligen* OR computer* OR sensor* OR detector* OR monitori* OR auto* OR remot* ) )

### Study eligibility

The literature was searched for all “smart” mandibular advancement devices for the treatment of OSA. A smart MAD was defined by the authors as an MAD that has any additional functionality besides protrusion of the mandible. Passive smart MADs were defined to be MADs that only collected patient data through sensors, while active smart MADs were defined to be those that responded automatically to data by altering the position of the jaw. Remotely controlled mandibular positioners (RCMPs), which are manually controlled live by a human, were decided not to be included in the definition of “smart.” All relevant articles mentioning such smart MADs were included. Articles written in languages other than English were excluded.

### Study selection

The bibliographic information of the articles found across the searched databases was retrieved and compiled by the information specialist (FJ). The titles and abstracts in the compiled list were imported in a web application for systematic reviews (Rayyan, Qatar Computing Research Institute, Doha, Qatar), and then reviewed by two reviewers (JY, BR) [15]. Articles for inclusion were selected independently by reviewers who were blind to each other’s selection. Screening decision conflicts were resolved during consensus meetings with two referees (JH, TR). The full texts of the included articles were retrieved for data registration by two independent reviewers (JY, BR) and the information specialist (FJ).

### Data registration and reported items

Two authors (JY, BR) used a web application (Google Sheets, Google Docs Editors, Google LLC, USA) to perform full-text screening and data registration of all the articles eligible for inclusion. A data extraction form, listed in supplement 1, was designed to record the information from the studies, as shown in summary in Table 4.

**Table 4:** Summary of data extraction form used for the articles on smart mandibular advancement devices (MADs). AHI = apnea-hypopnea index; RDI = respiratory disturbance index; SpO_2_ = peripheral capillary oxygen saturation. * - Type of study according to [add reference]. ** - TRLs according to [add reference]

Category	Data collected
Study and patient characteristics	- Country of first author - Type of study* - Study population and characteristics - Pre- and postoperative mean AHI - Pre- and postoperative mean RDI - Pre- and postoperative mean minimum SpO_2_ - Minimum and maximum follow up - Average preoperative BMI
Smart MADs	- Smart device purpose - System name - Parameters measured - Measurement validation - Sensor type - Technological readiness level**

The type of study was determined based on a modified version of the classification types as described by Röhrig et al. [16]. Studies were labeled as basic research if they included no patients; as observational if patients were included, but no intervention was carried out; and as interventional for clinical trials.

To determine the level of development, the technological readiness level (TRL), as introduced by the National Aeronautics and Space Administration (NASA), were used [17]. The nine TRLs are divided into the four phases listed in Table 5 [18]. The TRL for each device was interpreted by the authors.

**Table 5:** The technological readiness level (TRL) phases and definitions used by the authors to assign numerical values to the articles [17, 18]

Technological readiness level phase	TRL levels and definitions
Discovery	TRL 1, Basic principles of the technology are observed TRL 2, Technological concepts are formulated TRL 3, Proof of concept shown experimentally
Development	TRL 4, Prototypes validate proof of concept in laboratory TRL 5, Prototypes are tested in their relevant environment TRL 6, Prototypes are demonstrated but not optimized in their relevant environment
Demonstration	TRL 7, Prototypes are demonstrated in operational environment TRL 8, Technology is complete and validated
Deployment	TRL 9, Technology is commercially ready

## Results

**Fig. 1:**
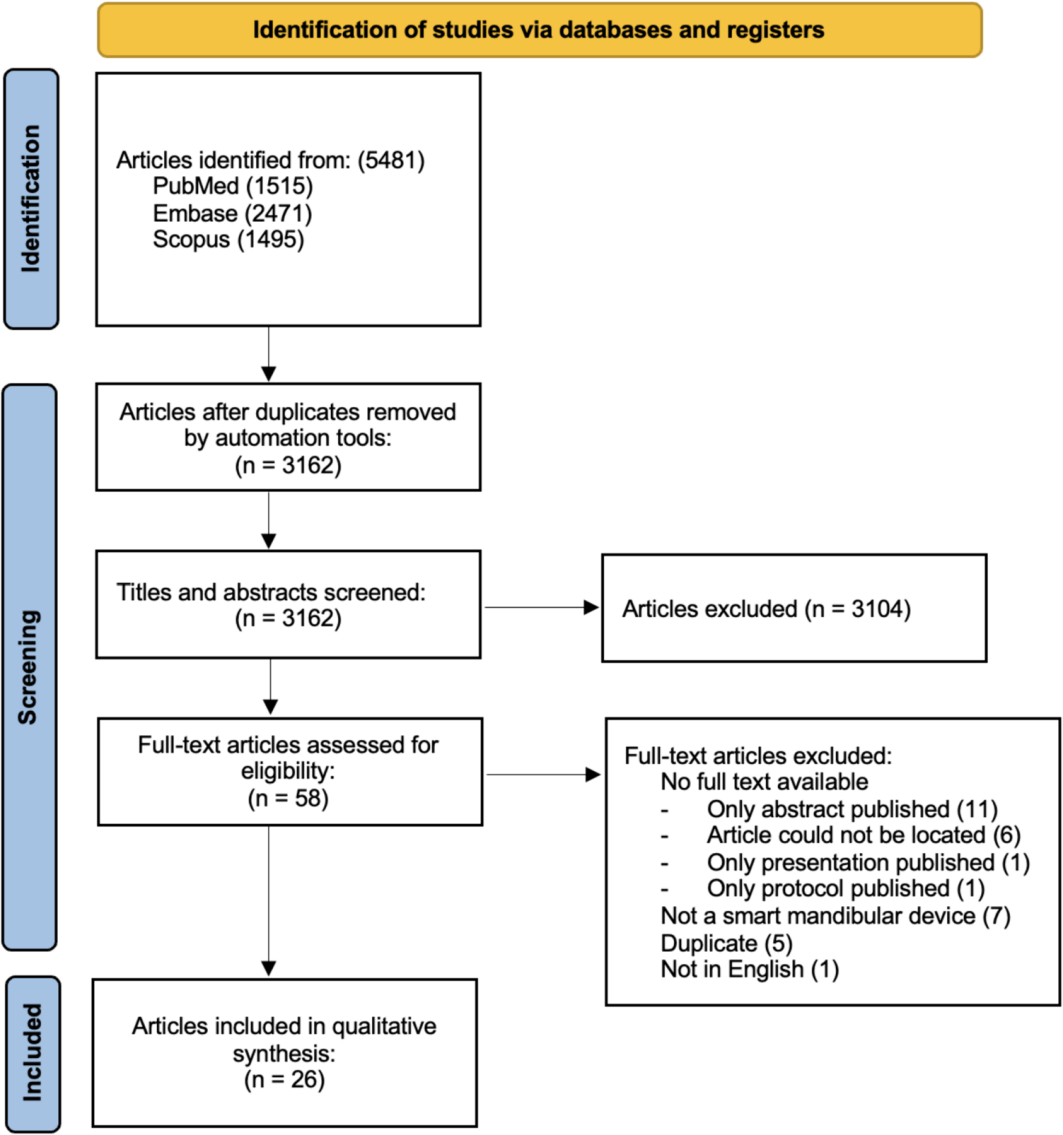
Diagram of the search and screening process

The literature search in the three databases yielded a total of 5481 articles (1515-PubMed, 2471-Embase, 1495-Scopus). Removal of duplicates left a total of 3162 articles eligible for title and abstract screening, which led to 58 articles to be assessed for full-text analysis. Articles were excluded for the reasons listed in Fig. 1, the most common reason being that a full text was not available (19 papers). Data were extracted by hand from the complete texts of 26 articles that were then included in qualitative synthesis.

### Literature and patient characteristics

The 26 included studies were divided into three categories: 21 were observational studies involving patients (81%); one was an interventional study (4%); and four were assessed as basic research (15%). All studies had been published between 2001 and 2022; the median year of publication was 2017.

The 22 patient-based studies (observational and interventional studies) included patients with OSA. Five (23% of articles with patients) used controls, who were either healthy controls or patients on CPAP therapy. In total, the articles included 1030 patients, the average study population per article being 46.8 patients (range: 1–180 patients). The average age of patients in the study groups was 50.1 years old (44.7–56.2 years old; 17/22 studies reporting). The average study group was 23.4% female (0–66.7%; 19/22 studies reporting). The average BMI of the study groups was 28.4 (21.3–34.3; 14/22 studies reporting). The average maximum follow-up time was 4.6 months (0.13–16.5; 15/22 studies reporting). All the mentioned averages in this paragraph are unweighted.

### Smart mandibular advancement devices

Smart MADs were grouped into two categories: those with passive functions and those with active functions, as presented in Table 6. Most smart MADs were passive (86%); their main functions included monitoring compliance (57%), monitoring therapeutic effect (18%), and collecting data solely for research purposes (11%). As some smart MADs performed multiple functions, Table 6 shows a higher number of articles than 26.

**Table 6:** Technological readiness levels (TRLs) of smart mandibular advancement devices, grouped by purpose and function. Passive MADs only record data, while active MADs respond by protruding the mandible. Some smart MADs perform more than one function

	Number of articles per purpose	DISCOVERY			DEVELOPMENT			DEMONSTRATION		DEPLOYMENT
Device Purpose		TRL 1	TRL 2	TRL 3	TRL 4	TRL 5	TRL 6	TRL 7	TRL 8	TRL 9
*Passive*										
To monitor compliance	16				[20]		[19]		[40, 41]	[11, 21–31]
To monitor therapeutic effect	5			[32]	[33–35]		[19]			
Other research purposes	3				[12]			[36]		[37]
*Active*										
Auto-adjusting	4						[10, 38]		[39]	[37]
Total number of articles per TRL (% out of 28)	28			1 (3%)	5 (18%)		4 (14%)	1 (3%)	3 (11%)	14 (50%)

#### Passive smart mandibular advancement devices

##### Monitoring compliance

Mandibular advancement devices that tracked compliance were described in 16 of the 26 articles (62%). Additionally, the greatest number of patients (in total 752 out of 1030) were found in articles studying MAD compliance.

All except two of the described smart MADs tracking compliance used thermosensors to measure intraoral temperature. One article [19] used a microphone to record snoring and respiratory sounds, and another [20] monitored compliance using a fiber optic sensor to measure tongue and splint pressure.

Most articles describing smart MADs tracking compliance have high levels of technological readiness, including several that are commercially available (TRL of 9) (Table 6). The three commercially available devices found in these studies were DentiTrac® (Braebon Medical Corporation, Kanata, Canada) [11, 21–25]; Theramon® (Orthosmart, Hoorn, Netherlands) [26–30]; and Thermocron™ (Measurement Systems Ltd, Newbury, United Kingdom) [31]. These smart MADs tracking compliance were found to be in the development, demonstration, and deployment (range of TRL levels: 4–9) stages of technological development, as displayed in Table 6.

##### Monitoring therapeutic effect

Mandibular advancement devices that monitored therapeutic effects other than compliance were described in five articles (19%). The sensors used in these devices included: microphones to record snoring and breathing sounds [19, 32]; pressure arrays to sense tongue-pressure distribution [32, 33]; accelerometers to register sleeping postures [34, 35]; and photoplethysmography (PPG) or SpO2 oscillators to measure cardiorespiratory parameters [34, 35]. These smart MADs were in earlier stages of technological readiness, i.e., in the discovery and development stages (range of TRL levels: 3–6), as shown in Table 6.

##### Other research purposes

Mandibular advancement devices were classified as performing research purposes if they were designed specifically for research, rather than being in development for clinical use. Such MADs were found in three articles (12%). The sensors used in them included: a force sensor used to measure forces applied by the arms of the MAD [36]; a microphone to record acoustical features of snoring while the MAD is worn [37]; and a pressure array to sense tongue-pressure distribution. These smart MADs were in the development, demonstration, and deployment (range of TRL levels: 4–9) stages of technological readiness, as shown in Table 6.

### Active smart mandibular advancement devices

Active smart MADs were described in four papers out of the 26 (15%) and included mandibular advancement devices that respond to data to adjust the position of the jaw, which are also known as feedback-controlled mandibular positioners (FCMPs). These devices were in the development, demonstration, and deployment (range of TRL levels: 6–9) stages of technological readiness, indicating that they were approaching or had already reached commercial availability.

The active MAD described by Brugarolas et al. [38] is named the Auto-Positioner and extends the mandible in response to two wearable sensors, a pulse oximeter and an accelerometer. The data is transferred to a computational node which receives and processes the sensor data, which then adjusts the jaw via a pump control; a magnetic field sensor then relays the degree of jaw extension. This article demonstrated a working prototype and was given a TRL rating of 6.

Remmers et al. [39] utilized a feedback-controlled mandibular positioner, which received signals regarding nasal airflow (from a dual catheter system) and oxyhemoglobin saturation (from a finger hood probe) on a laptop. The signals were processed and respiratory events were identified; a linear actuator would then move the mandible in response to the number and grade of respiratory events. A total of 179 patients were used to validate this smart MAD, and this technology was assigned a TRL rating of 7.

The only commercially available smart MAD described by the articles was the MATRx plus® (Zephyr Sleep Technologies, Calgary, Canada), which was used in two studies [10, 37]. The MATRx plus® is an in-home FCMP which repositions the mandible anteriorly-posteriorly based on instantaneous measurement of the oxygen desaturation index from polysomnographic observation. After attaching a microphone (Panasonic WM-61 A) to this MAD, Jacquet et al. [37] analyzed respiratory and snoring sounds. Mosca et al. [10] used this smart MAD to test the predictive accuracy of an artificial intelligence method against a heuristic, rule-based method. Because this technique was demonstrated with the MATRx plus® in a research environment, this smart MAD was assigned a TRL rating of 6.

## Discussion

### Summary of findings

The aim of this study was to provide clinicians, researchers, and manufacturers with a comprehensive, transparent, and evidence-based overview of the existing science and advancements in smart mandibular advancement devices (MADs). A total of 3162 articles were screened, 26 of which were included in the final qualitative synthesis. The median year of publication was 2017, thereby indicating that smart MADs are still a relatively new addition to this field of science. Most smart MADs were passive devices that tracked patient compliance, many of which use commercially available technology. A few active smart MADs were found in the literature, with one being commercially active. However, the level of evidence regarding the additional value of smart MADs over traditional MADs should be considered as low, since most of these studies were classified as observational (81%) or basic research (15%). Although this study was an extensive review, only one study [11] experimentally tested if wearing a smart MAD proved to be a more effective treatment option.

This review categorized smart MADs into two main categories: passive (monitoring compliance, monitoring therapeutic effects, other research purposes) and active function. Most articles (16 articles) concerned MADs that monitored patient compliance in wearing the device, and almost all of which used thermosensors to record intraoral temperature. Twelve of these studies used compliance monitors that were commercially available (TRL level of 9). The other passive functions of the smart MADs were monitoring of respiratory and snoring sounds, tongue pressure, accelerometer data, and oxyhemoglobin saturation. Some of these smart MADs were found to have been designed solely for the purpose of research, without the apparent intention of making them commercially available. Only four of the 26 articles described active smart MADs, or those that actively adjusted the protrusion of the jaw in response to patient health parameters. These MADs actively respond to health data. It has the potential to avoid unnecessary protrusion of the mandible and to minimize side effects such as temporomandibular joint complaints. However, there have yet to be clinical studies which demonstrate the effectiveness of these innovations for the treatment of OSA.

### Limitations

A comparatively large number of papers (19) were excluded due to the full text being unavailable for different reasons (Fig. 1). Also, no patent databases were consulted. Both might cause an underestimation of the development of (commercial) smart MADs. However, the authors are convinced that incorporation of these articles would not have significantly altered the conclusions of this review.

The definition of a “smart” mandibular advancement device (MAD), as implemented by the authors of this review, was ‘an MAD that has any other function besides protrusion of the mandible.’ This definition was chosen specifically to be as broad as possible to capture the widest range of articles pertaining to existing or developing smart devices. However, it can be discussed whether only active MADs in response to real time health monitoring should be considered as “smart”.

Another limitation of this paper is that certain characteristics of the devices were inferred by the authors, such as technological readiness levels or sensor characteristics, when they were alluded to but not directly stated in the articles. This may lead an interpretation error and an under- or overestimation of the technological readiness levels. Furthermore, as some articles derived from the same group of researchers publishing multiple papers on the same set of patients, this might lead to an overestimation of certain purposes of smart devices or sensors used.

### Research agenda

As smart mandibular advancement devices become more available and progress towards later stages of technological readiness, clinical research is crucial to test whether they have any measurable improved treatment outcomes. Preferably, new devices are compared with a control group consisting of patients treated with traditional, or “simple” mandibular advancement devices, for example with regards to a reduction in side effects or increase in compliance. A standardized set of patient-reported outcome measures (PROMs) would be helpful in this line of research. Additionally, the cost effectiveness of smart MAD treatment should be researched, as the cost of treatment might increase. Of further note, questions regarding the encryption, storage, and privacy of patient data should be answered as smart mandibular advancement devices become increasingly available. It should be made clear how this data is protected, and how it might be used for other purposes outside of research and patient improvement, such as for commercial and marketing purposes.

## Conclusion

This literature review provides the first evidence-based overview with innovations in MADs. It is the first to systematically update professionals in the field of OSA on the latest developments and discusses different opportunities for researchers. Smart MADs are a relatively new field in the treatment of OSA, but there are low levels of evidence demonstrating their additional value over traditional MADs. Most recently, initiatives regarding the development of “active” smart MADs have been described, in which protrusion of the mandible is adjusted to a variety of real time patient data. In the near future, smart MADs may have the potential to improve efficiency of OSA treatments and offer interesting new treatment opportunities.

## Electronic supplementary material

Below is the link to the electronic supplementary material.

Supplementary Material 1

## Data Availability

The authors declare that the data supporting the fndings of this study are available within the article.
